# PR genes of apple: identification and expression in response to elicitors and inoculation with *Erwinia amylovora*

**DOI:** 10.1186/1471-2229-6-23

**Published:** 2006-10-09

**Authors:** Jean M Bonasera, Jihyun F Kim, Steven V Beer

**Affiliations:** 1 Department of Plant Pathology, Cornell University, Ithaca, NY 14853, USA; 2 Present address: Laboratory of Microbial Genomics, Genome Research Center, Research Institute of Bioscience and Biotechnology, PO BOX 115, Yuseong, Daejeon 305-600, Republic of Korea

## Abstract

**Background:**

In the past decade, much work has been done to dissect the molecular basis of the defence signalling pathway in plants known as Systemic Acquired Resistance (SAR). Most of the work has been carried out in model species such as Arabidopsis, with little attention paid to woody plants. However within the range of species examined, components of the pathway seem to be highly conserved. In this study, we attempted to identify downstream components of the SAR pathway in apple to serve as markers for its activation.

**Results:**

We identified three pathogenesis related (PR) genes from apple, *PR-2*, *PR-5 *and *PR-8*, which are induced in response to inoculation with the apple pathogen, *Erwinia amylovora*, but they are not induced in young apple shoots by treatment with known elicitors of SAR in herbaceous plants. We also identified three *PR-1*-like genes from apple, *PR-1a*, *PR-1b *and *PR-1c*, based solely on sequence similarity to known *PR-1 *genes of model (intensively researched) herbaceous plants. The *PR-1*-like genes were not induced in response to inoculation with *E. amylovora *or by treatment with elicitors; however, each showed a distinct pattern of expression.

**Conclusion:**

Four PR genes from apple were partially characterized. *PR-1a*, *PR-2*, *PR-5 *and *PR-8 *from apple are not markers for SAR in young apple shoots. Two additional *PR-1*-like genes were identified through in-silico analysis of apple ESTs deposited in GenBank. *PR-1a*, *PR-1b *and *PR-1c *are not involved in defence response or SAR in young apple shoots; this conclusion differs from that reported previously for young apple seedlings.

## Background

Botanists have known for nearly 100 years that plants, like animals, can be immunized against pathogen attack by pre-treatment with another pathogen [1]. In the intervening years, many aspects of what is now referred to as Systemic Acquired Resistance (SAR) have been elucidated. The pathway leading to SAR involves three steps, pathogen recognition, signal relay and induction of genes, which facilitate synthesis of protective molecules. Once the pathogen is detected, the plant relays a signal through a complex network of signalling molecules to transcription factors that activate transcription of defence proteins or production of secondary metabolites [2]. Some downstream components have direct antimicrobial activity, while others work to restrict movement of the pathogen. Of those with direct antimicrobial activity, Pathogenesis-Related (PR) proteins have been used routinely in studies with model (intensively researched) species to assess the defence status of plants.

PR-proteins of plants have been defined as proteins of a host that are induced only in response to attack by pathogens or by a related event [3]. PR proteins are induced locally in response to pathogen attack as well as systemically in both compatible and incompatible host/pathogen interactions. Plants are able to coordinate, at the molecular level, the activation of expression of specific PR genes in response to attack by specific pathogens. For example, the suite of PR genes induced in *Arabidopsis thaliana *in response to the oomycete pathogen *Peronospora parasistica *differs from the suite induced in response to the fungus *Alternaria brassicicola *[4]. The precise role that most PR genes play in defense and in SAR has yet to be determined; however, expression of certain PR genes is coincident with development of resistance, and the induction/activation of PR genes is used routinely as a convenient marker of SAR [5].

There is a plethora of information about SAR and PR genes related to several model plants, especially, *Arabidopsis thaliana *[2], and members of the *Solanaceae *family, including tomato and tobacco [6,7]. In order for SAR to develop in these, plants must accumulate salicylic acid (SA). If SA is eliminated by the activity of an enzyme that hydrolyses it, resistance is not acquired [8]. Induction of *PR-1*, *2*, *5*, and *8 *is characteristic of SAR in several herbaceous plants. In tobacco, PR-1 protein can account for 1% of the total leaf protein in TMV-infected tissue [9]. In cucumber, *PR-8 *is robustly induced following treatment with SA or the related, but less phytotoxic compound INA (2,6-dichloroisonicotinic acid), both of which induce SAR [10].

Very little molecular evidence for SAR in woody perennials has been reported. Several groups have reported phenotypic resistance to pathogens following application of SAR elicitors such as SA or its functional analogs; benzo(1,2,3)thiadiazole-7-carbothioic acid-S-methyl ester (ASM) and INA to apple and pear [11-14]. However, none of these studies has demonstrated that the phenotypic resistance observed is the result of activating the SAR pathway. However, we hypothesized that this pathway occurs in apple because genes related to the pathway are highly conserved across the plant kingdom [9], including apple [15], and some components of the system share sequence similarity to proteins involved in innate immunity in the animal kingdom [16,17].

We undertook this study in an attempt to identify markers for the SAR pathway in apple. Specifically, we assayed apple tissue for induction of homologues of known PR genes following inoculation with the bacterial pathogen *E. amylovora*, which causes the devastating disease known as fire blight [18]. In addition, we assayed induction of PR genes in apple following treatment with known inducers of SAR in herbaceous plants.

## Results

### Identification of *PR-1a*, *PR-5 *and *PR-8 *from apple

The protein coding portions of three *PR *genes from apple were identified through a degenerate primed PCR approach with a cDNA library of *Malus *× *domestica *cv. Gala. The library, used as template in PCR, was developed from a pool of young apple shoots harvested from 0 to 6 days after inoculation with *E. amylovora *strain Ea273. Southern blot analysis of apple genomic DNA using the protein encoding regions of *PR-1a*, *PR-5 *and *PR-8 *from *Malus *× *domestica *cv. Gala as probes revealed that the three putative PR genes identified in apple, like those in other species, are members of multi-gene families. The full-length probes hybridized to multiple bands under high stringency conditions. Comparison of the predicted apple gene product to the type member for each group, as described by Van Loon et al[3], is shown in Table 1 here. The proteins from apple are similar in size, amino acid composition and isoelectric points to their respective type members.

The predicted gene products were analyzed for putative sub-cellular localization using PSORT, version 6.4, on the ExPASy Proteomics Server [19]. Apple PR-1a, PR-5 and PR-8 are predicted to have cleavable N-terminal signal sequences of 24, 24 and 20 amino acids, respectively. The protein products of the three apple genes identified are predicted to be secreted from the cell to the apoplast (Table 1).

The nucleotide sequences of apple *PR-1a*, *PR-5 *and *PR-8 *were deposited in GenBank [20], and the corresponding accession numbers are DQ318212, DQ318213 and DQ318214, respectively.

### Identification of three *PR-1 *genes from apple and their expression during flower development

**Figure 1:**
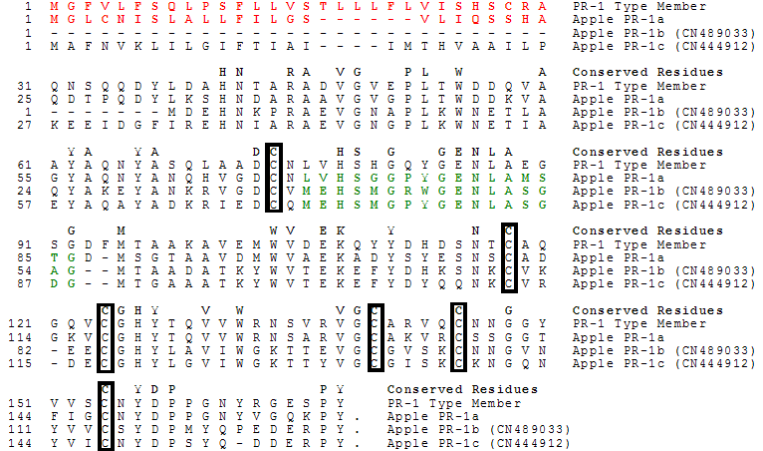
**Alignment of the deduced amino acid sequences of three apple *PR-1 *genes and the type member, PR-1a from tobacco (GeneBank:CAA29392)**. Residues shown in red are a predicted or known signal sequence. Boxed residues are the six conserved cysteine residues requisite in PR-1 type proteins. Residues shown in green were used in a tblastn query to generate data for table 2.

An in-silico analysis of apple ESTs deposited in GenBank was carried out to identify other members of the *PR-1 *family in apple. Three distinct groups of EST's were found based on predicted amino acid sequence similarity. The groups were arbitrarily designated *PR-1a*, *PR-1b *and *PR-1c*. An alignment of the three genes with the type member (tobacco *PR-1a*) is shown in Fig. 1. Each predicted apple protein contains the requisite six conserved cysteine residues that are present in the PR-1 family of proteins [21].

Of the three different apple *PR-1 *genes, the predicted protein product of *PR-1a *is most similar to the type member, tobacco PR-1a. Furthermore, *PR-1a *is the only PR-1 protein from apple reported to date that is predicted by PSORT to have a cleavable N-terminal signal sequence and to be localized outside of the cell (score = 0.820). PR-1c is predicted to contain an un-cleavable N-terminal signal sequence and to be localized to a membrane (plasma membrane score = 0.685; endoplasmic reticular membrane score = 0.640). PR-1b is predicted to be a cytoplasmic protein (score = 0.650).

In addition to predicted differences in sub-cellular localization, the three proteins have different patterns of expression as determined by in-silico analysis and confirmed by RT-PCR. The source tissue for apple ESTs corresponding to the *PR-1b *and *PR-1c *sequences was either fruit or flower tissue. In contrast, ESTs corresponding to *PR-1a *came from diverse sources; fruit (GenBank: CO576594), flower (GenBank: CO419366), shoot internode (GenBank: CV630152), leaf (GenBank: CV524932), bud (GenBank: CO903582) and even plantlets grown in-vitro (GenBank: AF507974) (Table 2).

**Figure 2:**
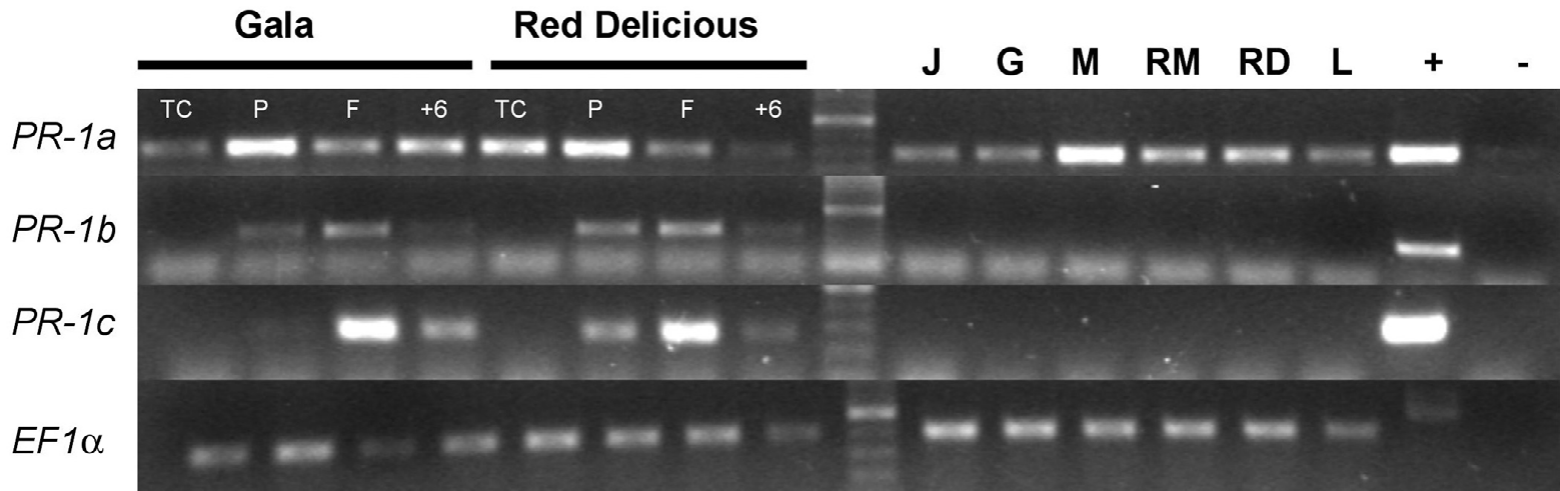
**Expression patterns of three different *PR-1 *genes from apple during flower development, and in several cultivars**. Two micrograms of total RNA was reverse-transcribed in a 20 μl reaction volume. Two μl of the resulting cDNA template from blossoms of cultivars Gala and Red Delicious at stages; tight-cluster (TC), pink (P), full-bloom (F) and 6 days post full-bloom (+6) or from shoots of the cultivars Jonagold (J), Gala (G), Mutsu (M), Rogers Mac (RM), Red Delicious (RD) and Liberty (L) were used in PCR with primers for PR-1a, PR-1b or PR-1c for 45 cycles. Ten μl of 25 μl reaction mixtures were loaded for each sample. For the EF1α control, 2 μl of the same cDNA template were amplified for 30 cycles with primers for EF1α. Ten μl of 25 μl reaction mixtures were loaded for each sample. Genomic DNA from cultivar Gala was used as the positive PCR control (+). The negative control (-) did not contain template. Note that the EF1α primers span an intron.

Based on in-silico analyses, the expression of *PR-1b *and *PR-1c *is restricted to flowers and fruits, while *PR-1a *transcripts are present in many different tissue types. These findings were supported by RT-PCR with primers specific for *PR-1a*, *PR-1b *or *PR-1c*. cDNA preparations from flowers at four stages of development from two apple cultivars, Gala and Red Delicious, were used as templates for PCR with specific primers. As determined by visualization of the PCR products in agarose gels, *PR-1a *transcripts were detected in both shoots and flowers of both cultivars with peak expression occurring during full bloom [22]. *PR-1b *transcripts were detected only in flowers of both cultivars with peak expression occurring between pink and full bloom. *PR-1c *transcripts also were detected only in flowers of both cultivars; peak expression occurred at the pink stage of flower development (Fig. 2).

### Inoculation with a florist's frog produces robust induction of *PR*-genes without inducing substantial expression of wound-response genes

**Figure 3:**
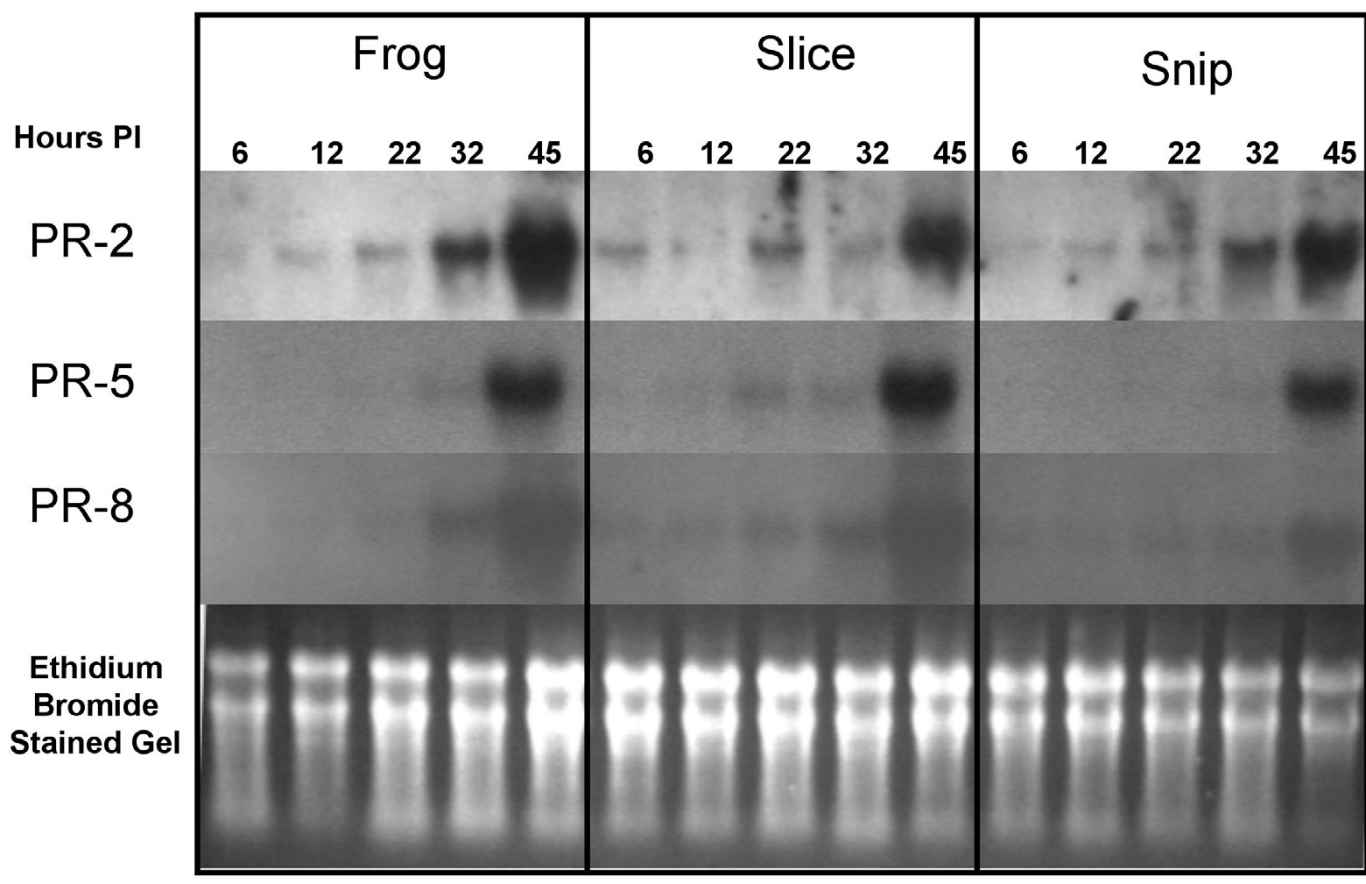
**Expression of *PR-2 *and *PR-5 *and *PR-8 *following inoculation of apple shoots with *Erwinia amylovora *by three different methods**. Northern hybridization of RNA preparations from young apple shoots following inoculation with *E. amylovora *Ea273 by piercing shoot tips with a contaminated florist's frog (**Frog**), slicing the two youngest unfolded leaves on either side of the mid-vein with contaminated scissors (**Slice**) or by snipping off the distal 1/3 of the two youngest unfolded leaves with contaminated scissors (**Snip**). Shoots or leaves were sampled at 6, 12, 22, 32 and 45 hours following inoculation.

Shoots of one-year-old *Malus *× *domestica *cv. Gala trees were inoculated with *E. amylovora *Ea273 using three different inoculation methods. PR genes were induced more rapidly in shoots of trees inoculated by puncturing leaves with the multiple pins of a florist's frog contaminated with bacteria, or by slicing both sides of the leaf parallel to the midvein with scissors contaminated with bacteria. The third inoculation method, snipping off the distal approximately 1/3 of the young leaf with contaminated scissors, proved to be the least robust method, and PR gene induction was delayed by 24 hours (Fig. 3). Both the frog and slice inoculation methods produced more severe disease symptoms than the snip inoculation method (data not shown).

### *PR-2*, *PR-5 *and *PR-8 *are induced in response to inoculation with *E. amylovora*

**Figure 4:**
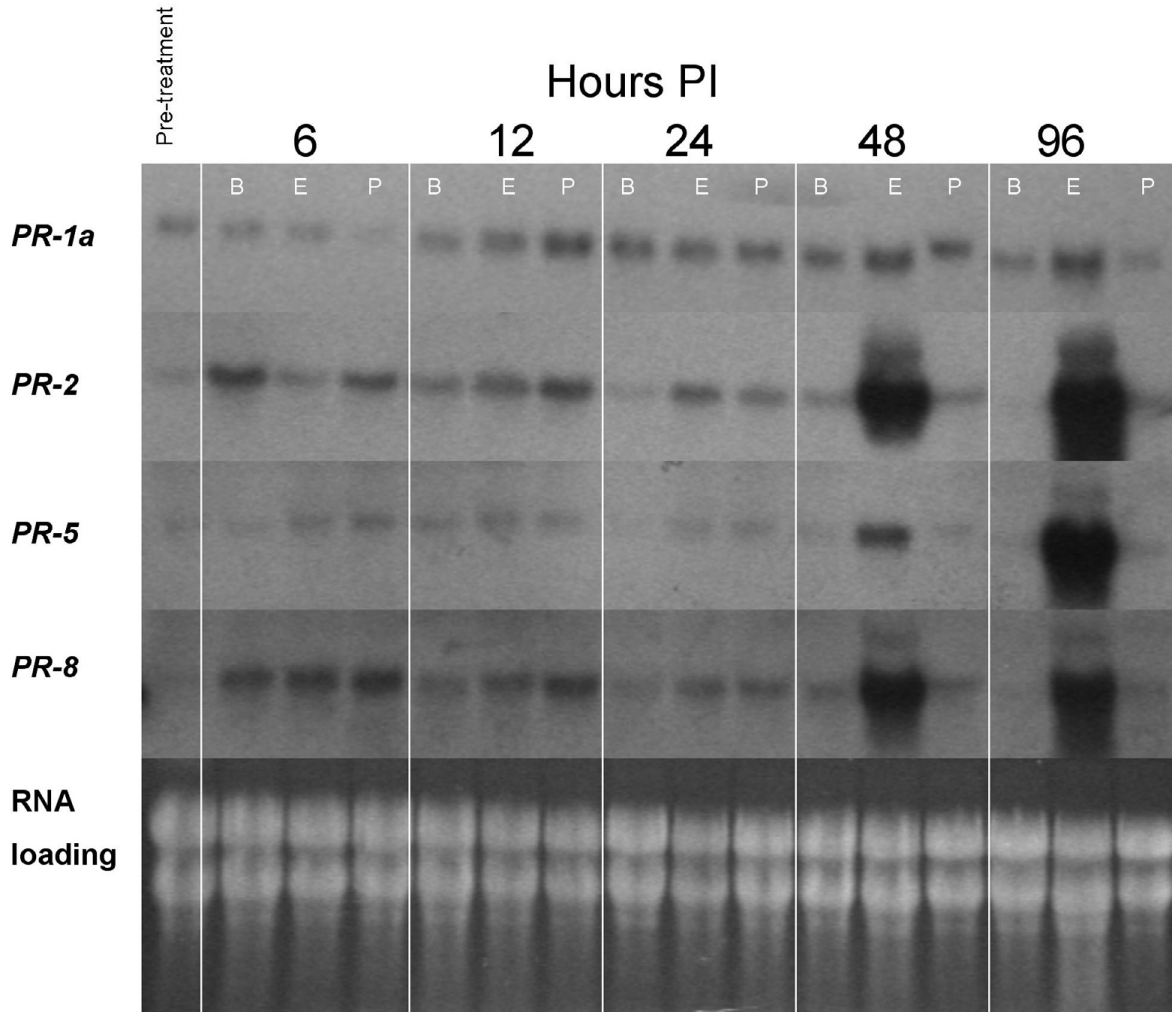
**Expression of apple PR genes in response to inoculation with plant pathogenic bacteria**. Northern hybridization of RNA preparations from young apple shoots just prior to (Pre-treatment), and following inoculation with *E. amylovora *Ea273 (**E**), *P. syringae *DC3000 (**P**), or mock inoculation with 5 mM potassium phosphate buffer pH 6.5 (**B**).

Northern hybridization studies were carried out with RNA isolated from apple shoots following inoculation with *E. amylovora *Ea273, *Pseudomonas syringae *pv. *tomato *(DC3000) or mock inoculation. Digoxigenin-labelled probes covering the entire open reading frames of *PR-5 *and *PR-8 *were used. In addition, a digoxigenin-labelled fragment of apple *PR-2 *(GenBank: AY548364) also was used as a probe. Expression levels were followed from pre-inoculation through 96 hours post-inoculation. *PR-2*, *PR-5 *and *PR-8 *were robustly induced in apple shoots between 24 and 48 hours post-inoculation with *E. amylovora*, but expression of *PR-2*, *PR-5 *and *PR-8 *was not induced in either mock-inoculated or *P. syringae*-inoculated apple shoots (Fig. 4).

### *PR-1a *is not induced in response to inoculation with *E. amylovora*

In contrast to the robust induction of *PR-2*, *PR-5 *and *PR-8*, *PR-1a *was not induced during the first 96 hours following inoculation of young apple shoots with *E. amylovora *Ea273. In addition, *PR-1a *was not induced in tissues in response to inoculation with *P. syringae *DC3000 (Fig 4). The expression level of *PR-1a *remained constant during the first 96 hours following inoculation with the compatible pathogen, Ea273, the non-pathogen, *P. syringae *DC3000 or mock-inoculation. Furthermore, no expression of *PR-1b *or *PR-1c *was observed in apple shoots following inoculation with *E. amylovora*, as determined by RT-PCR using a pool of RNA's purified from apple shoots harvested 0 to 6 days post inoculation as template (data not shown).

### *PR-1a*, *PR-2*, *PR-5 *and *PR-8 *are not induced in response to treatment with elicitors

**Figure 5:**
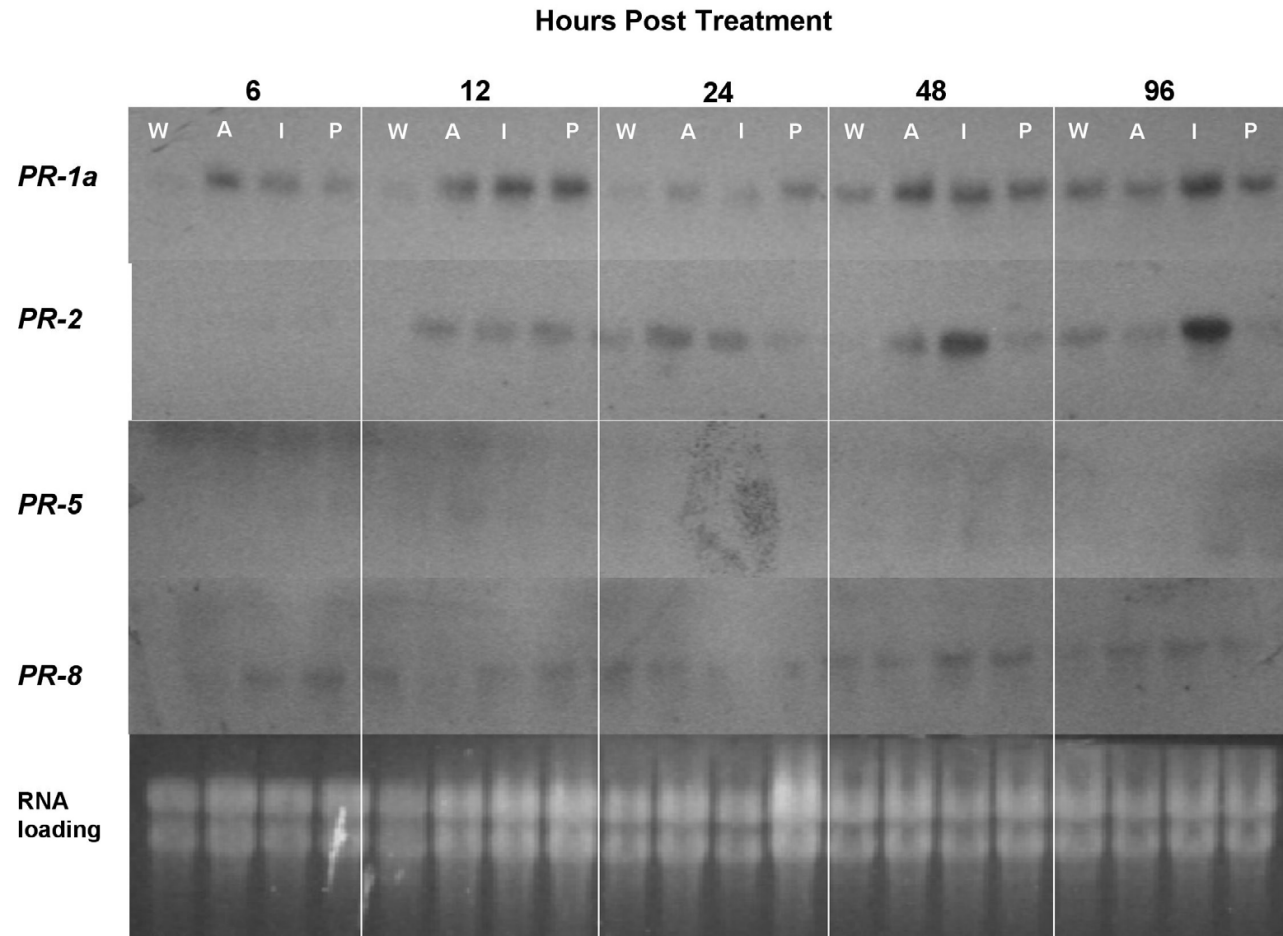
**Expression of apple PR genes in response to treatment with SAR elicitors**. Northern hybridization of RNA preparations from young apple shoots following spray application of water **(W)**, Actigard^® ^**(A)**, INA **(I) **or ProAct^® ^**(P)**. 15 μg of total RNA was loaded in each lane.

None of the four apple PR genes identified here were induced during the first 96 hours following treatment with ASM or ProAct^®^, as determined by northern hybridization analysis (Fig. 5). Subtle induction of *PR-2 *observed between 48 and 96 hours after spraying shoots with INA could be a wound response since INA applied at 250 mg active ingredient (AI) per liter proved phytotoxic to apple leaves and shoots within 48 hours after spray application.

## Discussion

We identified four genes as candidates for involvement in the response of apple to attack by *E. amylovora *based on their similarity to genes documented as involved in SAR in other plants. Three of the four apple genes, *PR-2*, *PR-5 *and *PR-8*, but not *PR-1a*, conform strictly to the definition of a PR gene described by Van Loon et al. [3]; they are up-regulated in response to inoculation with the pathogen, *E. amylovora*.

**Figure 6:**
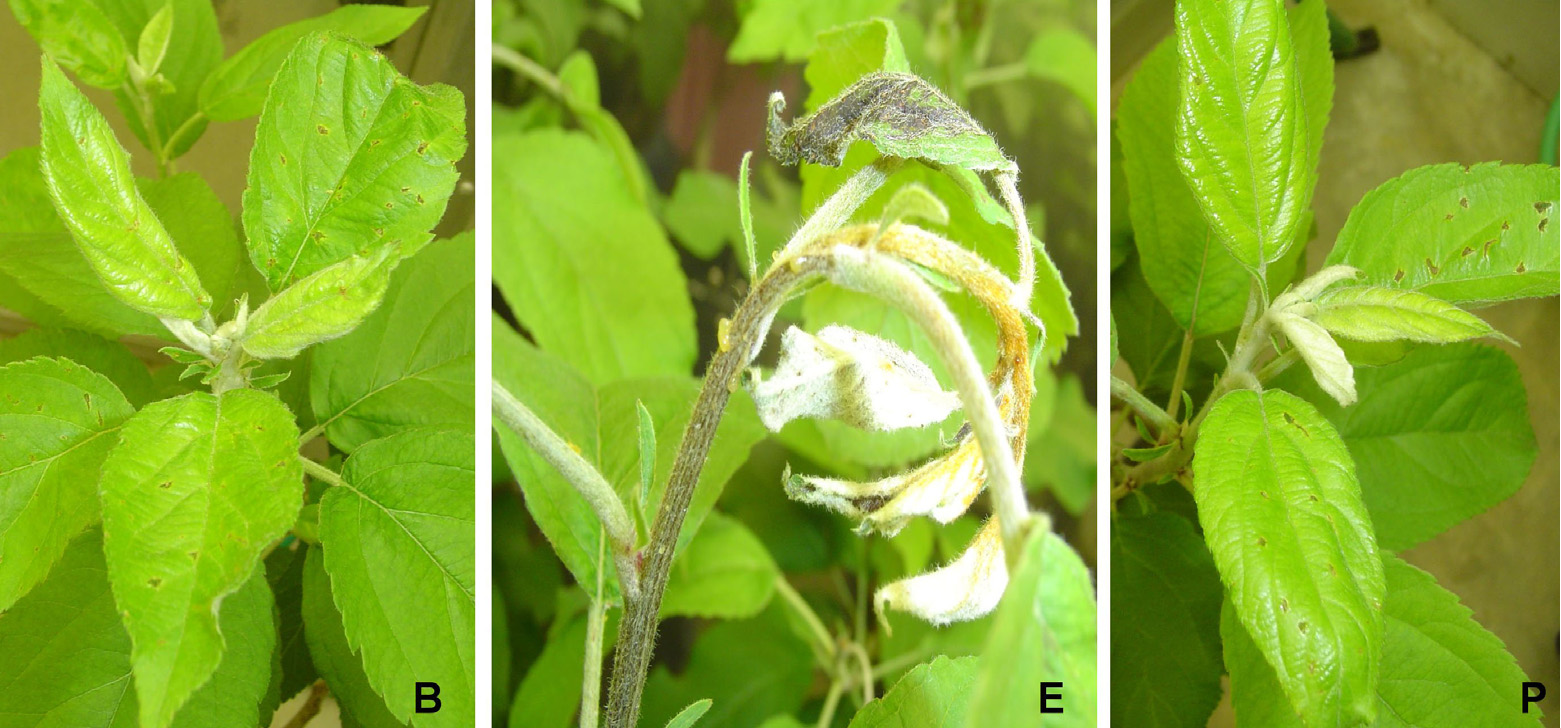
**Phenotype of apple shoots 96 hours following inoculation**. Apple shoots were either mock-inoculated (**B**), or inoculated with *E. amylovora *Ea273 (**E**) or *P. syringae *DC3000 (**P**). Note the wounds made by the inoculating pins. Wounds are not evident in **E **because the inoculated leaves were totally necrotic when photographed.

We were surprised that *PR-1a *was not induced following inoculation with the apple pathogen, *E. amylovora*. Based on work in Arabidopsis, tobacco and other species [9], we expected apple to readily produce every defense protein in its arsenal, including PR-1 given the degree of tissue damage present by 96 hours after inoculation (Fig 6). The apple PR-1a protein identified here clearly fits into the family of PR-1 proteins; its sequence predicts that it should be secreted from plant cells, and it is similar to the PR-1 proteins from other species that are involved in pathogen interactions. Thus, based on our studies in apple shoots, inoculated with *E. amylovora*, *PR-1a *falls short of meeting the strict definition of a PR gene, and may be more properly referred to as a "PR-like" gene.

The other two members of the PR-1 gene family identified here, *PR-1b *and *PR-1c *diverge significantly from *PR-1a *in the highly conserved fourth alpha helix region. They are expressed in distinctive patterns during flower development; they were not expressed in apple shoots whether or not the shoots were inoculated with *E. amylovora*. This is an interesting observation, which raises the question as to the possible involvement of PR-1b and PR-1c in floral development.

Although we cannot rule out the possibility that an unidentified member of the *PR-1 *gene family exists in apple, which is up-regulated during pathogen interactions, a recent report by Gau et al. [23] seems to support our conclusion that *PR-1 *is not induced in apple shoots during pathogen attack. These authors analyzed the protein content of apoplastic fluid of the apple cultivar Elstar following inoculation with *Venturia inaequalis*, the apple scab pathogen. They did not detect any PR-1-type protein up to 21 days following inoculation. Thus, for at least two apple pathogens, *E. amylovora *and *V. inaequalis*, *PR-1 *is not part of an induced defence response in shoots for at least the first 96 hours and 21 days following inoculation, respectively.

In 2004, Sparla et al. reported a study in which they had treated pear trees, another important host of *E. amylovora*, with 10 mM SA or ASM at 200 mg AI per liter [13]. Trees were challenged with *E. amylovora *10 days later. There was a significant reduction in disease incidence and severity in treated trees. However, expression of *PR-1 *was not affected by treatment of pear shoots with ASM or SA or following inoculation with *E. amylovora*; the authors concluded that *PR-1 *was expressed constitutively in pear shoots and was likely not involved in SAR in pear [13].

Several other groups have reported increased resistance to the development of fire blight in host plants treated with ASM [11,12,14]. Maxson-Stein et al. demonstrated resistance to fire blight in orchard-grown apple trees and PR gene induction in apple seedlings following spray application of ASM at 250 mg AI per liter [11]. Brisset et al. demonstrated resistance to fire blight in 2-year-old greenhouse-grown apple trees and increased chitinase and glucanase activity in apple seedlings following treatment with ASM at 200 mg AI per liter [14]. Ziadi et al. demonstrated systemic as well as local induction of apple *PR-10 *in apple seedlings following spraying with ASM at 200 mg AI per liter [24]. In each of these studies, gene expression analyses were carried out using apple seedlings; however, the resistance phenotype was observed in much more mature woody trees. In the work reported here, application of Actigard^® ^at 250 mg AI per liter to apple shoots growing on mature wood did not result in significant induction of the four PR genes assayed (*PR-1a*, *PR-2*, *PR-5*, *PR-8*). The dose of Acitigard^® ^used in this study was well within the range used by others, and is more than 10 times the application rate recommended in the product literature [25]. The difference in results might be due to the developmental state of the treated tissue; apple seedlings may respond differently to elicitor treatment than young shoots growing on mature wood. Even so, in comparison to the levels of gene induction seen in Arabidopsis and tobacco, where the SAR pathway has been well studied, meaningful induction of PR genes in apple in response to treatment with elicitors of SAR is questionable, at best.

Our studies of PR gene expression in shoots following treatment of 1-year-old apple trees with elicitors do not support the conclusion that induction of the SAR pathway is responsible for the phenotypic increase in resistance to fire blight reported by others [11,12,14]. In contrast to Arabidopsis and tobacco, in which PR genes are rapidly and robustly induced following treatment with elicitors [7,26], none of the four PR genes we identified in apple were induced in apple shoots during the first 4 days following treatment with elicitors. We believe that the modest induction of *PR-2 *we observed following treatment with INA at 250 mg AI per liter was a wound response coincident with the development of phytotoxicity.

We evaluated three methods for inoculating shoots of 1-year-old apple trees with *E. amylovora *with respect to extent and rate of symptom development and for induction of PR gene expression. The florist's frog method is similar to a method used by van der Zwet and Keil [27], but it involves more individual points of inoculation. The method seems to rather closely mimic one of the means by which shoot inoculation occurs in orchards. Shoot infection often is initiated following traumatic events experienced by young growing shoots, through the activity of insects, wind-driven rain or hail. The second method, slicing the young leaf lamina on both sides of the mid-vein, was used to try to maximize the number of plant cells exposed to the bacterium at time zero. The third method, snip, a standard method of inoculation [28], was included as a bridge to previous work. Trees inoculated using either the florist's frog or the slice method showed symptoms sooner and induced PR genes more rapidly than the snip method. The florist's frog and slice methods seemed equivalent with respect to PR gene induction and the severity and rate of development of disease symptoms. We chose to use the florist's frog method as our standard method of inoculation because it seemed to more closely approximate natural infection than the slice method. In addition, use of the florist's frog is rather straight forward and inoculation is rapidly accomplished. Also, unlike the snip method, the florist's frog immediately exposes a large number of plant cells to bacteria, thus it likely facilitates a better picture of the early events following recognition of *E. amylovora *by apple cells.

## Conclusion

Enhanced expression of *PR-2*, *PR-5 *and *PR-8 *was apparent in apple shoots 24 to 48 hours after inoculation with *E. amylovora*, the fire blight pathogen. Enhanced expression of *PR-2*, *PR-5 *and *PR-8 *was not observed when apple shoots were inoculated similarly with *P. syringae *pv. *tomato*, a non-pathogen of apple.

The expression of *PR-1a *in apple shoots was not enhanced during the first 96 hours after inoculation with either *E. amylovora *or *P. syringae *pv. *tomato*, nor was *PR-1a *expression induced in response to treatment with compounds known to elicit SAR in other plants. Thus, we conclude that *PR-1a*, *PR-1b *and *PR-1c *are not involved in defence response or SAR in young apple shoots; this conclusion differs from that reported previously for young apple seedlings.

Treatment of apple shoots with elicitors of SAR in other plants did not result in enhanced expression of any of the four PR genes identified in apple. Thus, we were not able to identify markers for SAR in apple.

Inoculation of apple shoots with the pins of a florist's frog contaminated with cells of *E. amylovora *was effective in inducing expression of PR genes; symptom development occurred rapidly following inoculation with the florist's frog.

## Methods

### Plant materials

Dormant 1-year-old *Malus *× *domestica *cv. Gala trees were planted in soil mix (1 part Cornell mix: 1 part Agway^® ^Potting Soil (Southern States Cooperative, Inc. Richmond, VA USA) : 1 part Perlite with Osmocote (Scotts Miracle-Gro Co., Marysville, OH USA) in 3.8-liter pots and placed in the greenhouse. Trees were trained to two shoots. When shoots were 20–30 cm long, the trees were transferred to a controlled environment chamber where they were maintained at 24°C – 26°C with a 12-hour photoperiod (380 μM/m^2^s incandescent and fluorescent) and a minimum relative humidity of 65% for the remainder of the experiment. Trees were given a 3 – 4 day equilibration period in the growth chamber prior to further manipulation.

Apple flowers, staged according to Chapman and Catlin [22], were harvested directly into liquid nitrogen from trees growing in an orchard near Ithaca, NY. Flowers were held at -80°C or colder until RNA was isolated, as described below for shoots.

### Bacterial inoculations

*Erwinia amylovora *strain Ea273 or *P. syringae *pv. *tomato *(DC3000) were grown for 16 hours at 26°C on plates of Luria-Bertani (LB) medium. Colonies were transferred to 5 mM potassium phosphate buffer, pH 6.5, using a cotton swab. The density of the suspension was adjusted to O.D._600 _= 0.2, which corresponded to 10^8 ^cells/ml. Unless mentioned otherwise, inoculations were performed between 2 and 4 hours into the light cycle by dipping a florist's frog (4.8 cm in diameter with 127 pins) into freshly prepared inoculum and then puncturing the fanned-out shoot tip held against a nitrile-gloved hand. The dip and puncture procedure was repeated once. Mock inoculation was similar except that 5 mM potassium phosphate, buffer pH 6.5 was used rather than bacterial suspensions. For the inoculation optimization study, the first two unfolded, but unexpanded leaves, of ten shoots of apple trees were cut either perpendicular or parallel to the mid-vein with scissors or were punctured twice with the pins of a florist's frog dipped in inoculum. Two shoots representing each inoculation method were collected at each time point.

### Elicitor treatment

Elicitors were sprayed to run-off using a hand-pumped atomizing sprayer. Elicitors were diluted in water and were applied 2 to 4 hours into the light cycle. INA was applied at 250 mg AI per liter. ASM, as Actigard^® ^(Syngenta Crop Protection, Greensboro, NC USA), was applied at 250 mg AI per liter. ProAct^® ^(Eden Bioscience, Bothell, Washington USA) was applied at 15 mg AI per liter.

### RNA manipulations for northern hybridizations

Harvested apple shoots were frozen by plunging the excised portions into liquid nitrogen. Once frozen, the tissue was stored at -80°C. RNA was isolated from the leaf tissue as described by Komjanc et al. [29], then quantified using the Quant-iT™ RiboGreen^® ^RNA Assay Kit, as directed by the manufacturer, (Molecular Probes, Inc. Eugene, OR USA).

Fifteen micrograms of total RNA was resolved through a denaturing gel as described by Sambrook et al. [30]. The gel was stained with ethidium bromide and photographed after electrophoresis. The resolved RNA was transferred to an uncharged nylon membrane (Cat. No. N00HYB0010, GE Osmonics Labstore, Minnetonka, MN USA) using a phosphate buffer-based transfer system [31]. RNA was fixed to the membrane by baking as directed by the manufacturer. Membranes were hybridized to probes covering a 723-bp fragment of apple *PR-2 *(GenBank:AY548364), or the entire open reading frames of apple *PR-1a*, *PR-5 *and *PR-8 *(Table 1). Probe labelling and hybridization conditions were as directed in the PCR DIG Probe Synthesis Kit (Roche Molecular Biochemicals, Indianapolis, IN, USA). Detection was carried out as directed by the manufacturer using the chemiluminescent substrate, "CSPD, ready-to-use" (Roche Molecular Biochemicals).

### PCR protocols

Degenerate primers were designed based on alignment of several known PR gene sequences deposited in GenBank. First, the degenerate primers were used to amplify putative PR gene fragments from genomic *Malus *× *domestica *cv. Gala DNA. The amplicons were sequenced on an ABI 3700 DNA Sequencer at the Cornell University Biotechnology Resource Center Sequencing Facility. Specific primers were designed using the primer select program from DNASTAR, based on the sequences obtained from the degenerate primed amplicons. Finally, apple PR gene-specific primers were used in combination with vector-specific primers to amplify the entire open reading frames from a cDNA library of shoots of 1-year-old *Malus *× *domestica *cv. Gala trees harvested from 3 hours to 6 days following inoculation with *E. amylovora *strain Ea273 as described above using the snip method. The library was constructed using the SMART cDNA Synthesis kit (Clontech, Palo Alto, CA, USA) following the LD PCR protocol. The full-length open reading frame (with the exception of *PR-2*, with which attempts to amplify a full-length open reading frame were unsuccessful) amplicons were cloned into pBluescript II KS+ (Stratagene, La Jolla, CA, USA) and sequenced. PCR was carried out using either Pfu Turbo^® ^(Stratagene) or DyNAzyme™ EXT (Finnzymes Oy, Espoo, Finland) DNA polymerase, dNTP's (Promega), primers (Integrated DNA Technologies, Coralville, IA USA or Cornell University Biotechnology Resource Center, Ithaca, NY USA). An annealing temperature of 55°C was used for all primer sets except *PR-1b*; primers were given 1 minute per kb amplicon for extension at 72°C. An annealing temperature of 50°C was used for *PR-1b*. Cycle number was optimized for each template and primer combination, as noted in the figure legends.

### Southern hybridization

Genomic DNA was isolated from three cultivars of apple – Jonagold, Gala and Roger's Mac, using the procedure described by Dellaporta et al. [32]. Ten micrograms of genomic DNA was digested with *Eco *RV or *Hind *III, resolved on an agarose gel and transferred to uncharged nylon membranes [30]. Membranes were probed as described above for northern hybridizations.

### RT-PCR

Two micrograms of total RNA were reverse transcribed as described by Wilson and Melton [33], except that random hexamers (Promega, Madison, WI, USA) were used in place of oligo dT to prime the RT reaction. PCR was carried out as described above. The primers used are listed in Table 3.

### Bioinformatics

DNA sequence analysis, protein deduction, statistics and alignments were generated using Lasergene^® ^from DNASTAR (Madison, WI, USA). Protein localization prediction analysis was run through PSORT [19]

## Authors' contributions

JMB carried out the experiments, conducted the in-silico analyses, prepared the figures and drafted the manuscript. JFK was instrumental with degenerate primer design and manuscript revision. SVB was responsible for experimental design and revised and polished the manuscript. All authors have read and approved the final manuscript.

## Figures and Tables

**Table 1:** Side-by-side comparison of three putative PR proteins from apple with their respective type member.

	**Apple PR-1a**	**PR-1 Type Member CAA29392**	**Apple PR-5**	**PR-5 Type Member CAA27548**	**Apple PR-8**	**PR-8 Type Member AAC37395**
**Similarity to Type Member E-value^1^**	5e-54	N/A	2e-37	N/A	2e-104	N/A
**Molecular Weight (Daltons)**	17122	18574	25669	24552	31757	30775
**Amino Acid Residues**	161	168	246	226	299	292
**Basic Amino Acids**	8	10	14	14	16	13
**Acidic Amino Acids**	17	14	20	16	24	24
**Hydrophobic Amino Acids**	55	48	75	63	109	115
**Polar Amino Acids**	61	54	89	86	102	91
**Isoelectric Point**	4.718	5.803	4.567	5.248	4.600	4.273
**Charge at pH 7.0**	-8.296	-3.473	-6.405	-2.435	-7.798	-10.766
**Number Amino Acids in Signal Sequence**	24	30	24	25	20	25
**Predicted Sub-cellular Location**	Outside	Outside	Outside	Outside	Outside	Vacuole

^1 ^The E (expect) value is the probability that the match happened by chance. Comparison was made with mature peptide sequences (i.e. without signal sequence)

Deduced amino acid sequence statistics of *PR-1a*, *PR-5 *and *PR-8 *from apple were generated using the Editseq program in Lasergene^® ^from DNASTAR (Madison, WI, USA). Protein sequences for type members were obtained through GenBank and analyzed using the same program. Sequence similarities to type members were obtained by using the BLAST on the National Center for Biotechnology Information web site. Signal sequence and localization predictions were done by PSORT. The type members are as described by Van Loon et al. [3]

**Table 2:** In-silico comparison of the deduced amino acid sequence of three *PR-1 *type genes from *Malus *× *domestica*

**Parameter**	**PR-1a**	**PR-1b**	**PR-1c**
**Signal sequence**	24 aa Cleavable	None	19 aa Un-cleavable
**Predicted Location**	Outside of the cell	Cytoplasm	Plasma membrane
**Leaf Accessions**	10	0	0
**Shoot Accessions**	1	0	0
**Bud Accessions**	2	0	0
**Flower Accessions**	4	5	5
**Fruit Accessions**	4	2	1

The deduced amino acid sequences of three different PR-1 type genes from apple were analyzed for their sub-cellular localization using PSort. The number of accessions in GenBank and their source tissue was obtained by tblastn query of National Center for Biotechnology Information Genbank data base using the 17-amino-acid sequence denoted in green in Figure 1.

**Table 3:** Primers used for RT-PCR and probe synthesis

**Gene name**	**Primer Sequence (5' → 3')**
*PR-1a*	gctcagccgtaatacaatcctctc tacccccactactgcacctcact
*PR-1b*	gtttgctgcgcccattag ttgcactttgaaacaccacatc
*PR-1c*	agcttattttgggcatcttcacc gtagttttgccccatatcacacca
*PR-2*	cttcacagtcaccatcttcaaca ggtgcaccagctttttcaa
*PR-5*	ggcaggcgcagttccaccag gacatgtctccggcgtatca
*PR-8*	caaaaacggcaatgaaggaacc ctggcgagctcatcatagaactgc
*EF1α*	agaccaccaagtactactgcac ccaccaatcttgtacacatcc
